# Circular RNA mediated gene regulation in human breast cancer: A bioinformatics analysis

**DOI:** 10.1371/journal.pone.0289051

**Published:** 2023-07-26

**Authors:** Giulia Fiscon, Alessio Funari, Paola Paci

**Affiliations:** 1 Department of Computer, Control and Management Engineering, Sapienza University of Rome, Roma, Italy; 2 Institute for Systems Analysis and Computer Science “Antonio Ruberti”, National Research Council, Rome, Italy; 3 Department of Translational and Precision Medicine, Sapienza University of Rome, Roma, Italy; BMSCE: BMS College of Engineering, INDIA

## Abstract

Circular RNAs (circRNAs) are a new acknowledged class of RNAs that has been shown to play a major role in several biological functions both in physiological and pathological conditions, operating as critical part of regulatory processes, like competing endogenous RNA (ceRNA) networks. The ceRNA hypothesis is a recently discovered molecular mechanism that adds a new key layer of post-transcriptional regulation, whereby various types of RNAs can reciprocally influence each other’s expression competing for binding the same pool of microRNAs, even affecting disease development. In this study, we build a network of circRNA-miRNA-mRNA interactions in human breast cancer, called CERNOMA, that is a bipartite graph with one class of nodes corresponding to differentially expressed miRNAs (DEMs) and the other one corresponding to differentially expressed circRNAs (DEC) and mRNAs (DEGs). A link between a DEC (or DEG) and DEM is placed if it is predicted to be a target of the DEM and shows an opposite expression level trend with respect to the DEM. Within the CERNOMA, we highlighted an interesting deregulated circRNA-miRNA-mRNA triplet, including the up-regulated hsa_circRNA_102908 (BRCA1 associated RING domain 1), the down-regulated miR‐410-3p, and the up-regulated ESM1, whose overexpression has been already shown to promote tumor dissemination and metastasis in breast cancer.

## Introduction

Circular RNAs (circRNAs) are a special class of non-coding RNAs that are generated by a process of non-canonical splicing that joins a 5’ splice site to an upstream 3’ splice site, resulting in a covalent closed loop [1–3]. CircRNAs are widely observed in both plants [4] and animals [5], and even if their biological functions remain broadly unknown, increasing evidence suggests them as crucial regulators of multiple biological processes, including the development and progression of human diseases such as cancers [6–13]. The high resistance to degradation of circRNAs, which is dependent on their circular structure, makes them different from other linear RNAs. This stability causes tissues such as blood and plasma to be especially enriched with circular RNAs compared to messenger RNAs (mRNAs) and other non-coding RNAs [14]. Thus, when released into the bloodstream by tumoral cells, circRNAs can be more easily detected with respect to other transcripts, revealing them as good potential biomarkers for early diagnosis, metastasis, and prognosis [15]. Several findings reported that circRNAs are aberrantly modulated in human cancer tissues, thus affecting carcinogenesis and metastatization, and can also be useful for predicting and monitoring treatment response [12, 16, 17]. Even though no circRNA have been effectively used as biomarkers in clinical trials yet, the impact of circRNA-mediated regulation on various cell transcriptome showed a great potential to be investigated especially in human diseases [14, 18]. Interestingly, recent studies have been focusing on the possibility that circRNAs can operate as part of competing endogenous RNA (ceRNA) regulatory networks, playing major roles in normal development and in pathologic conditions like human cancer [12, 15, 18–25].

The ceRNA mechanism is a recent discovery providing a possible explanation of fine-tuned post-transcriptional gene regulation orchestrated by the competing endogenous RNAs and microRNAs (miRNAs) [26–30]. microRNAs are small non-coding RNAs (∼ 20–22 nucleotides long) responsible for RNA silencing and post-transcriptional regulation of gene expression [31]. The ceRNA hypothesis states that various types of RNAs can reciprocally influence each other’s expression competing for binding the same pool of miRNAs, thus preventing mRNAs to be targeted [26]. This RNA-RNA crosstalk can add a new level to the understanding of complex regulatory networks that, when perturbed, could lead to disease development [19, 32–35].

Among several computation tools for ceRNAs discovery, we recently developed SPINNAKER [36], the R-implementation of the well-established model [37] that was acknowledged as the best one in terms of percentage of identified RNAs acting as ceRNA in breast cancer tissues [38]. By exploiting a multivariate statistical analysis, SPINNAKER first searches for highly correlated RNA pairs (i.e., co-expressed) and then evaluates the extent to which this correlation is direct or mediated by miRNAs, via the computation of the sensitivity correlation [37]. Finally, SPINNAKER selects only those RNA pairs whose interaction is mediated by some miRNAs (i.e., highest sensitivity correlation) and builds a ceRNA network where nodes are ceRNAs and links are miRNAs mediating their interactions. The ceRNA network can be optionally refined by considering only those triplets with ceRNAs showing a predicted binding site for the miRNA.

**Fig 1:**
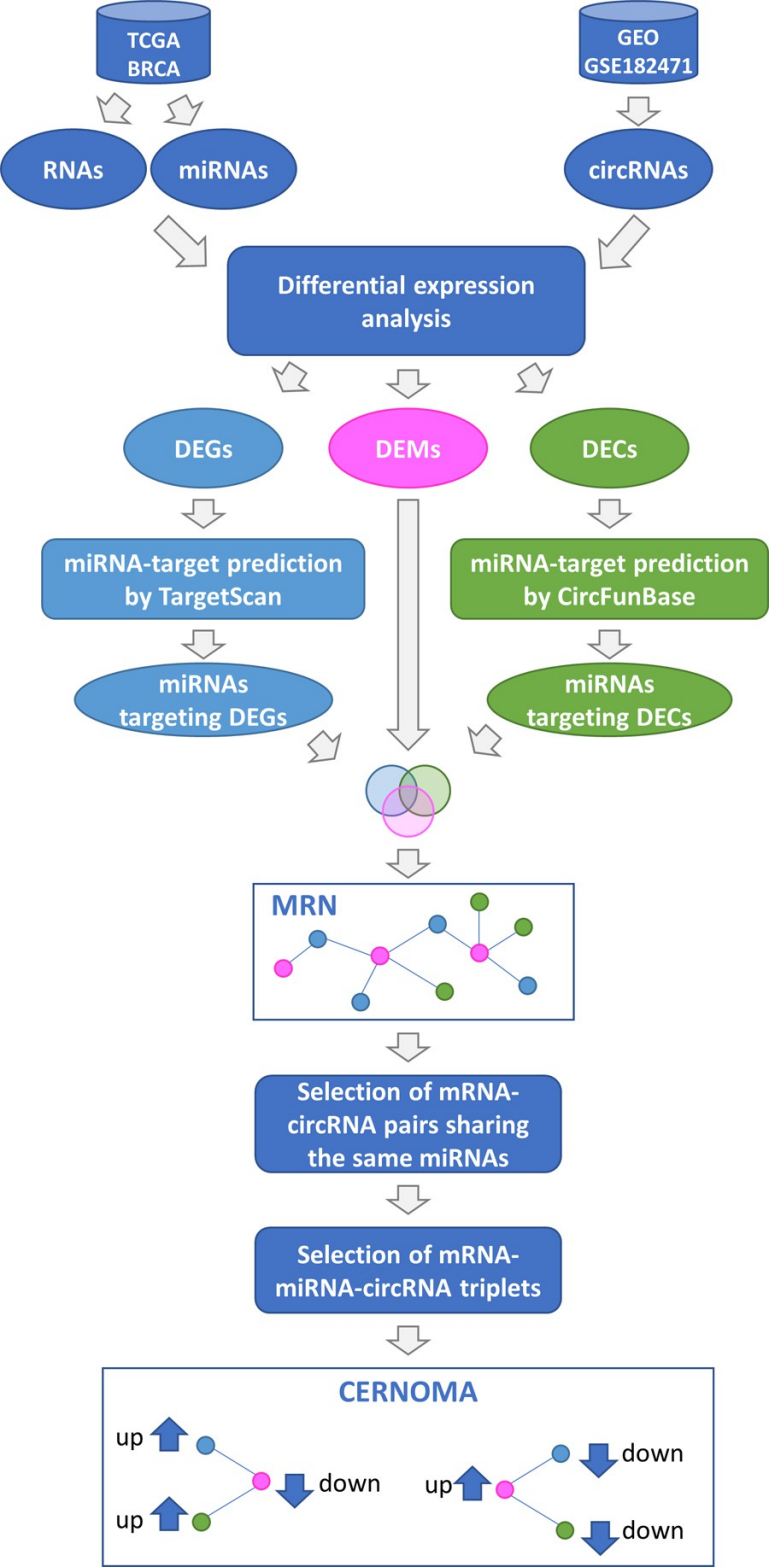
Workflow of the study. The input data are: (i) RNA- and miRNA-sequencing expression data from breast invasive carcinoma (BRCA) and matched-normal tissues retrieved from TCGA, (ii) microarray data of circRNAs from breast cancer and adjacent no-tumor breast tissues retrieved from GEO (GSE182471). Expression data were processed to obtain differential expressed RNAs (DEGs), differential expressed miRNAs (DEMs), and differential expressed circRNAs (DECs) between normal and breast cancer tissues. Next, the miRNAs predicted to target DEGs and DECs were obtained from TargetScan and circFunBase, respectively. The predicted miRNAs and the DEMs were intersected and a miRNA-target regulatory network (MRN) was constructed. Then, mRNA-circRNA pairs sharing the same miRNA and showing an opposite expression level trend with respect to the miRNA were retained. The so-called CERNOMA network was thus obtained and released as final output of the analysis.

To run SPINNAKER and build the ceRNA network, we need as input three matrices of RNA expression levels from the same cohort of tissues/cells (i.e., two matrices for the two classes of candidate competing RNAs and one for the miRNAs). Unfortunately, these types of data are not always available, especially for the recently acknowledged class of circRNAs. To tackle this issue, in this study we developed a new computational pipeline to unveil the regulatory role of circRNA in the miRNA-target interaction network (Fig 1), when we are unable to apply SPINNAKER.

First, we build a miRNA-target regulatory network (MRN), where nodes are miRNAs and their target genes (in this case are circRNAs and mRNAs) being significantly differentially expressed between normal and cancer tissues. Then, we generate its mapping onto the space of ceRNA network, ending up with the here-defined CERNOMA. The CERNOMA network is obtained from the MRN: (i) by selecting only the circRNAs and mRNAs sharing the predicted binding site for the same miRNAs, and (ii) by narrowing the circRNA-miRNA-mRNA triplets to those ones with a specific expression pattern. Specifically, we selected those triplets whose mRNA and circRNA show the same expression level direction (significantly up- or down-regulated) and whose miRNA shows an opposite direction (significantly down- or up-regulated). This selection should mirror the action provided by SPINNAKER to retain only the highly correlated pairs with a highest sensitivity correlation, when the correlation and thus the sensitivity correlation cannot be computed.

By applying the pipeline to study breast invasive carcinoma, within the CERNOMA network we can identify some circRNAs modulated in breast cancer exhibiting a putative regulatory activity with respect to other RNAs.

## Materials and methods

### Expression data collection

High throughput RNA-sequencing and miRNA-sequencing expression data of breast invasive carcinoma (brca) were acquired from The Cancer Genome Atlas (TCGA) data portal on February 2022 [39]. RNA-sequencing data correspond to read counts calculated by HT-Seq and FPKM normalized. miRNA-sequencing data correspond to normalized counts in reads-per-million-miRNA-mapped. A total of 204 samples, 102 tumor and 102 matched-normal tissues (i.e., the matched-normal tissue is defined as the tissue that is adjacent to the tumor and taken from the same patient) for both RNA- and miRNA-sequencing experiments were retained for the subsequent analysis.

Microarray dataset providing circular RNA (circRNAs) expression profile data, detected with 074301 Arraystar Human CircRNA microarray V2 on August 2021, from 5 breast cancer tissues and 5 adjacent non-tumor breast tissues were acquired from Gene Expression Omnibus (GEO) [40] database (GSE182471).

### Differential expression analysis

Collected expression data were first analyzed by performing the following two phases, following the same procedure implemented in [41–43]:

#### Pre-processing

Expression data were first processed by applying a logarithmic (log2) transformation and then were filtered out those genes having too many missing values among the samples (i.e., we filtered out entries showing missing values for more than 75% of the samples) and those genes with a little variation—measured by the Inter Quartile Range (IQR) percentile—across the samples (i.e., we filtered out entries showing an IQR lower than the 10^th^ percentile of the IQR distribution).

#### Filtering

The logarithmic ratio of the average expression levels of tumor samples and matched-normal samples (log fold-change) was computed and those genes falling behind, in absolute value, a fixed cutoff on the log fold-change were removed. Then, according to the type of samples distribution, a parametric (Student’s t-test for RNAs and miRNAs) or non-parametric (Wilcoxon test for circRNAs) statistical test was performed. Finally, the obtained p-values were independently adjusted for each type of data set by using False Discovery Rate (FDR) method and those genes showing an FDR lower than a chosen cut-off were considered as statically significant.

At end of this step, the differentially expressed RNAs (DEGs), the differentially expressed miRNAs (DEMs), and the differentially expressed circRNAs (DECs) between tumor and normal samples were obtained.

### miRNA-target regulatory network

Starting from DEGs, DECs, and DEMs, a miRNA-target regulatory network is built via the following two phases:

#### Prediction of miRNA-target interactions

Predictions of miRNAs targeting the differentially expressed mRNAs were obtained by querying TargetScan [44], which is the most up-to-date tool providing computationally predicted miRNA-mRNA interactions by searching for the exact matching between the seed region of a miRNA and the 3’ UTR of its targets.

Predictions of miRNAs targeting the differentially expressed circRNAs were obtained by querying circFunBase [45], which is a comprehensive database of functionally annotated circRNAs with more than 7000 functional circRNA entries regularly updated with newly published data, and including also computationally predicted miRNA-circRNA interactions.

Names and features for circRNAs and miRNAs refer to circBase [46] and miRBase [47] sources, respectively.

#### Network construction

**Fig 2:**
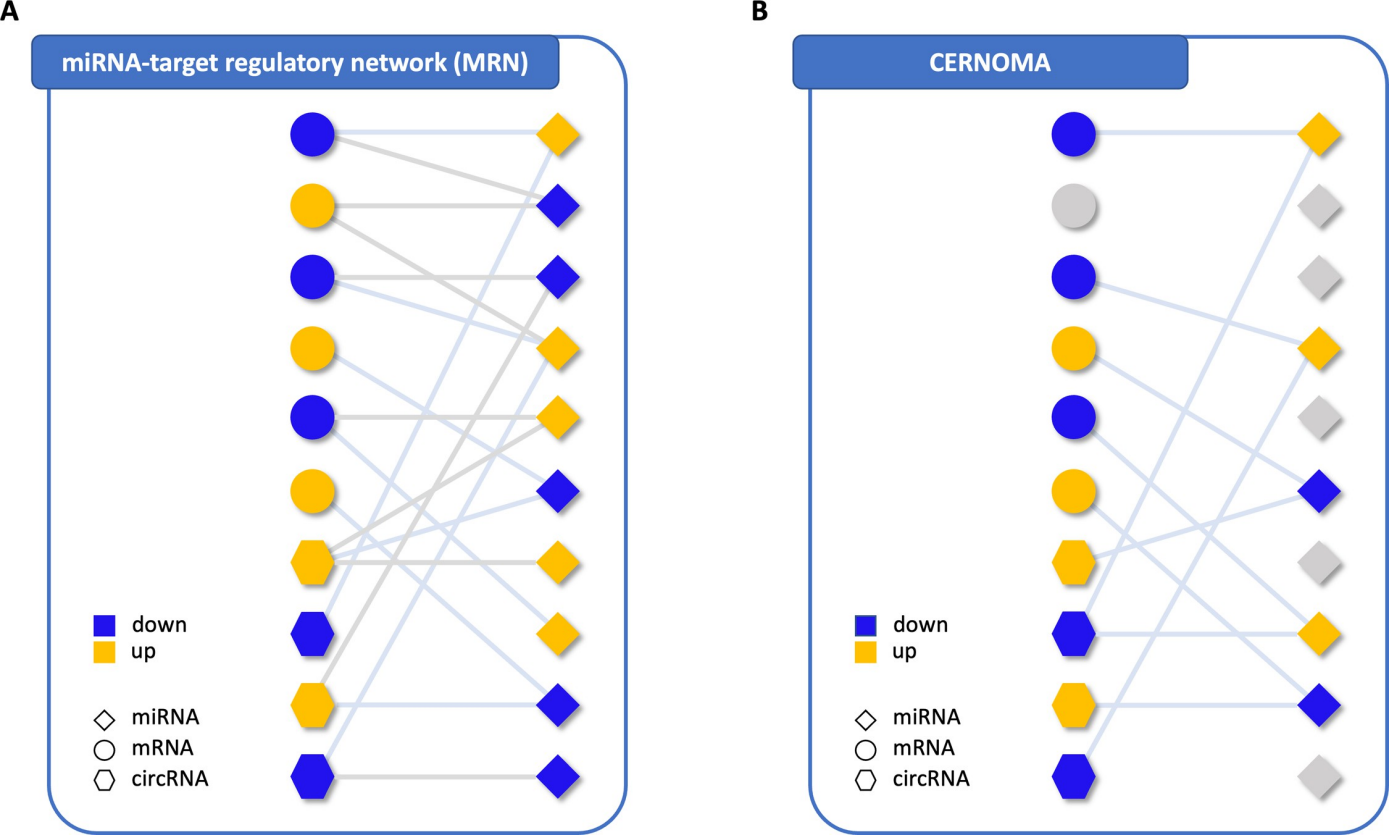
Regulatory network sketches. A) miRNA-target Regulatory Network (MRN). It is a bipartite network, where one class of nodes corresponds to differential expressed miRNAs (diamond), the other one corresponds to differential expressed circRNAs (octagons) or differentially expressed mRNAs (circles), a link between them occurs if a circRNA or mRNA is predicted to be target of the miRNA. **B) CERNOMA.** It is the mapping of MRN onto a ceRNA space. It is a bipartite network with the same classes of MRN nodes, but a link between them occurs (light blue color) both if the circRNA and mRNA are predicted to be target of the same miRNA and they show an opposite expression level trend with respect to the miRNA targeting them (up-regulated circRNA and mRNA, down-regulated miRNA or *viceversa*). Yellow and blue nodes refer to up- and down-regulated genes in breast cancer tissues, respectively. Grey nodes refer to unselected nodes when mapping MRN onto CERNOMA. Grey links refer to links of MRN that are not in CERNOMA, light blue links refer to links of MRN that are mapped in the CERNOMA.

The miRNAs predicted to target DECs and DEGs were then intersected with DEMs and a miRNA-target regulatory network (MRN) was constructed as a bipartite network, where one class of node corresponds to DEMs and the other one corresponds to DECs or DEGs. A link between them is placed if a DEC or DEG is predicted to be target of the same DEM (Fig 2A).

At the end of this step the miRNA-target regulatory network is released.

### CERNOMA

The miRNA-target regulatory network is finally mapped onto a ceRNA space, where first the circRNAs and mRNAs sharing the same miRNAs were selected. Then, following the expression direction suggested by a ceRNA mechanism, the circRNA-miRNA-mRNA triplets were filtered in order to retain only those ones where the miRNA showed an opposite expression level trend with respect both to the circRNA and mRNA predicted to be its targets (i.e., up-regulated miRNA, down-regulated circRNA and mRNA or *viceversa*). Thus, the so-called CERNOMA was built as a bipartite network, where one class of nodes corresponds to DEMs and the other one corresponds to DECs and DEGs. A link between them is placed if both the DEC and DEG are predicted to be target of the DEM and show an opposite expression level trend with respect to the DEM (Fig 2B).

At the end of this step the CERNOMA is released and draw by using Cytoscape software [48].

### Functional enrichment analysis

enrichR software [49] was used to perform Gene Ontology (GO) analysis and Kyoto Encyclopedia of Genes and Genomes (KEGG) pathway enrichment analysis about the differentially expressed genes appearing in the CERNOMA network that were targets of at least one differentially expressed miRNAs. An adjusted p-value ≤ 0.05 was set as threshold to identify significantly enriched functional annotations amongst the selected gene list.

## Results

### Differential expression analysis

**Fig 3:**
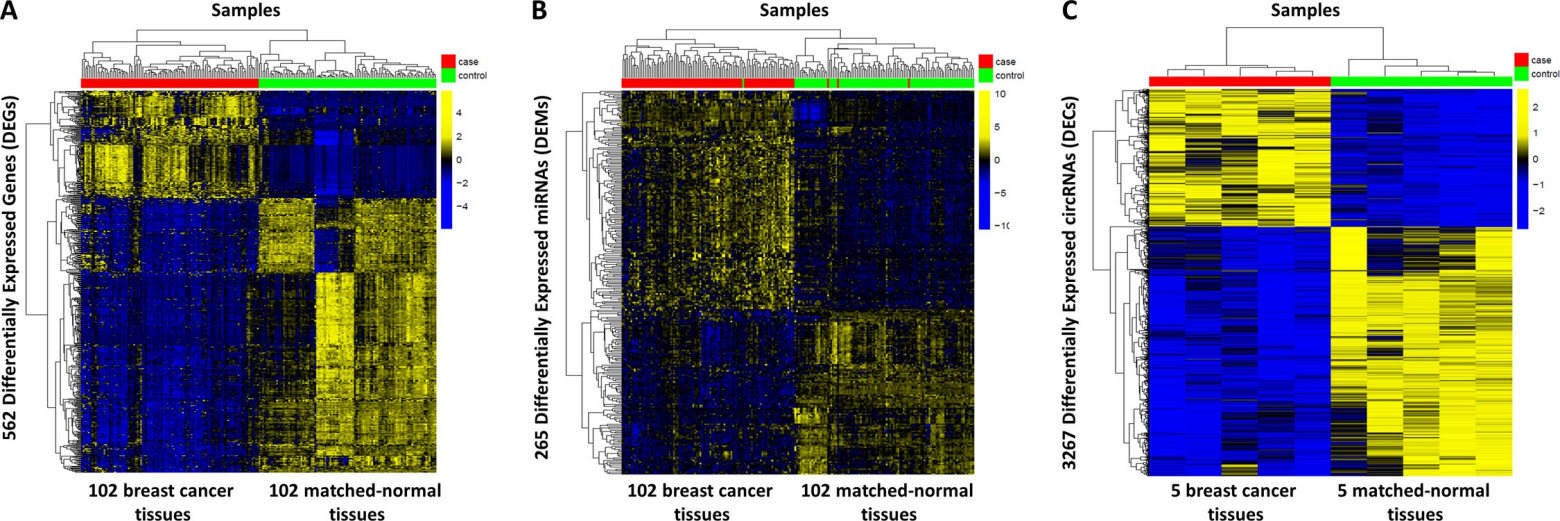
Heatmap and dendrogram of (A) differentially expressed RNAs (DEGs), (B) differentially expressed miRNAs (DEMs), and (C) differentially expressed circRNAs (DECs). The expression profiles of DEGs, DEMs, and DECs are clustered according to rows (genes) and columns (samples) by using as distance metrics 1-*ρ*, where *ρ* is the Pearson correlation and complete linkage algorithm as clustering method. Heatmap colors represent different expression levels (z-score normalized) that increase from blue to yellow. Red bars refer to breast cancer tissues, while green bars refer to matched-normal breast tissues.

RNAs, miRNAs, and circRNAs expression data were first pre-processed and then analyzed by conducting a differential expressed analysis in order to extract genes that were significantly deregulated in breast cancer tissues (cf. Materials and Methods). According to the parameter settings defined in Table 1, we obtained a total of 562 DEGs, 265 DEMs, and 3267 DECs (S1 Table), whose expression profiles are able to clearly discriminate between breast cancer and non-tumoral adjacent breast tissues, as observed by the well-defined hierarchical clustering (Fig 3).

**Table 1:** Summary of differential expression analysis thresholds and results. DE (Differentially expressed), FC (Fold-change).

	Adjusted p-value threshold	FC threshold	# of DE	# of UP	# of DOWN
**RNAs**	0.05	3	562	158 (28%)	404 (72%)
**miRNAs**	0.05	1.5	265	150 (57%)	115 (43%)
**circRNAs**	0.1	1.5	3267	1164 (36%)	2103 (64%)

### miRNA-target regulatory network

To investigate the ability of DECs and DEGs to bind miRNAs, we searched for predictions of miRNA-mRNA and miRNA-circRNA interactions by querying TargetScan and circFunBase database, respectively. We thus obtained a total of 39 miRNAs predicted to target 10 DECs and 231 DEGs. We then retained only those 17 miRNAs that were also differentially expressed in breast cancer and we constructed a miRNA-target regulatory network as a bipartite network composed of ten circRNAs (3 down-regulated and 7 up-regulated), 302 mRNAs (223 down-regulated, 79 up-regulated), 136 miRNAs (71 down-regulated, 65 up-regulated), and 2107 edges (S2 Table).

### CERNOMA

In order to identify a putative RNA-RNA cross-talk in breast cancer tissues, starting from the miRNA-target regulatory network (MRN), we generated the CERNOMA, i.e., the MRN mapping onto a ceRNA space, where circRNAs and mRNAs share the same miRNA and are characterized by opposite expression levels trend with respect to the miRNA predicted to target both of them. The CERNOMA shows a total 208 circRNA-miRNA-mRNA triplets, and it is composed of 218 miRNA-mRNA/circRNA interactions (edges), four circRNAs (2 up-regulated and 2 down-regulated in breast cancer tissues), ten miRNAs (8 up-regulated and 2 down-regulated in breast cancer tissues), and 103 mRNAs (12 up-regulated and 91 down-regulated in breast cancer tissues) (S2 Table).

The basic features of the four circRNAs modulated in breast cancer and appearing in the CERNOMA were summarized in Table 2.

**Table 2:** Main features of circRNAs appearing in the CERNOMA, retrieved from circFunBase [45].

circRNA	Gene symbol	Gene description	Location hg19 (strand)	UP/DOWN in brca
hsa_circRNA_407041	MSR1	macrophage scavenger receptor 1	chr8:16353301–16372347 (-)	DOWN
hsa_circRNA_104342	BBS9	Bardet-Biedl Syndrome 9	chr7:33185853–33217203 (+)	DOWN
hsa_circRNA_102908	BARD1	BRCA1 associated RING domain 1	chr2:215632205–215646233 (-)	UP
hsa_circRNA_403236	ZNF827	zinc finger protein 827	chr4:146767107-146824367(-)	UP

**Fig 4:**
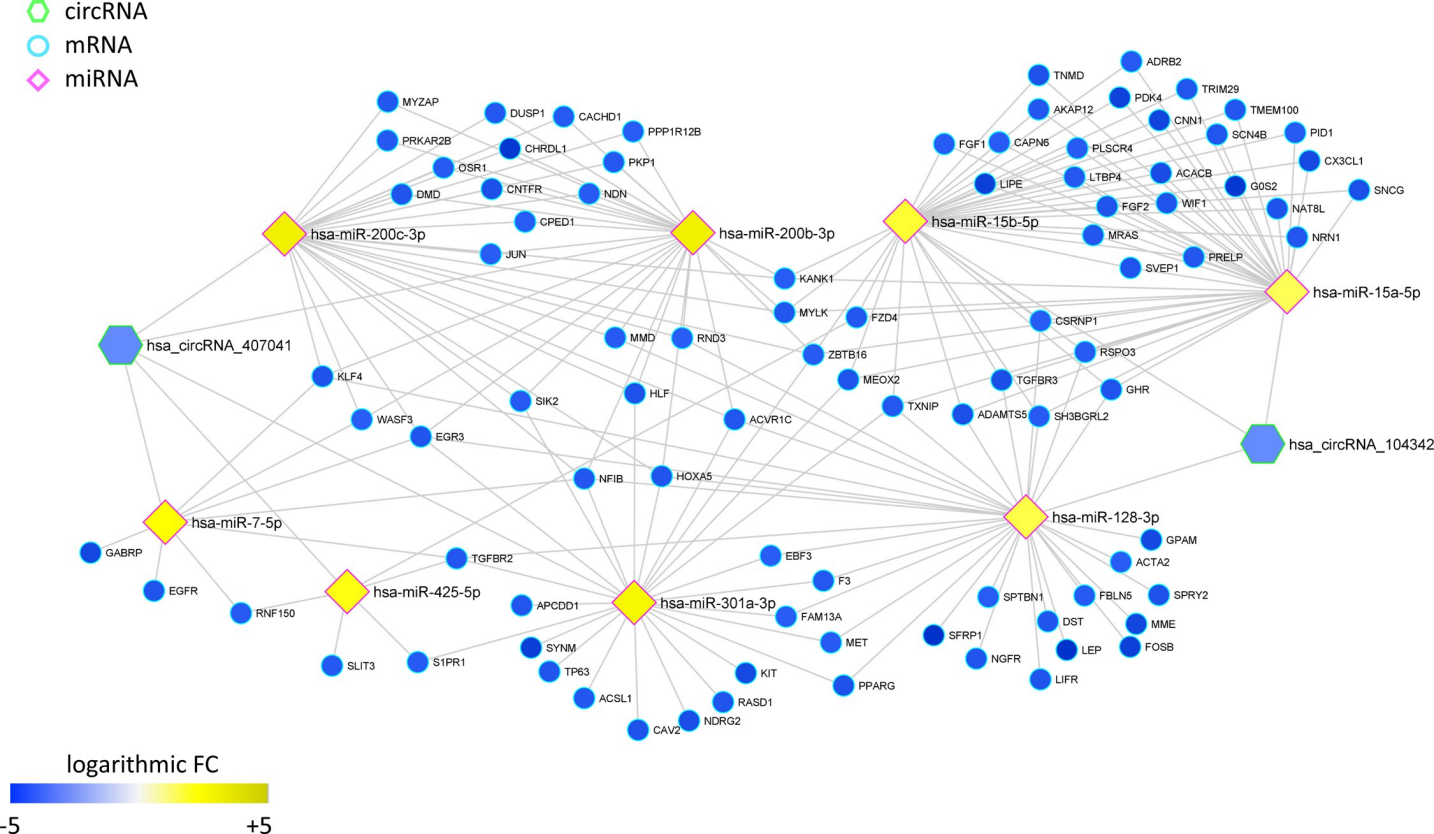
First largest connected component of CERNOMA for breast cancer dataset. Network showing the circRNA-miRNA-mRNA interactions. Diamonds represents miRNAs, octagons represent circRNAs, circles represent mRNAs. Gradual changes in node color represent differences in the expression levels of different genes (increasing from blue to yellow).

**Fig 5:**
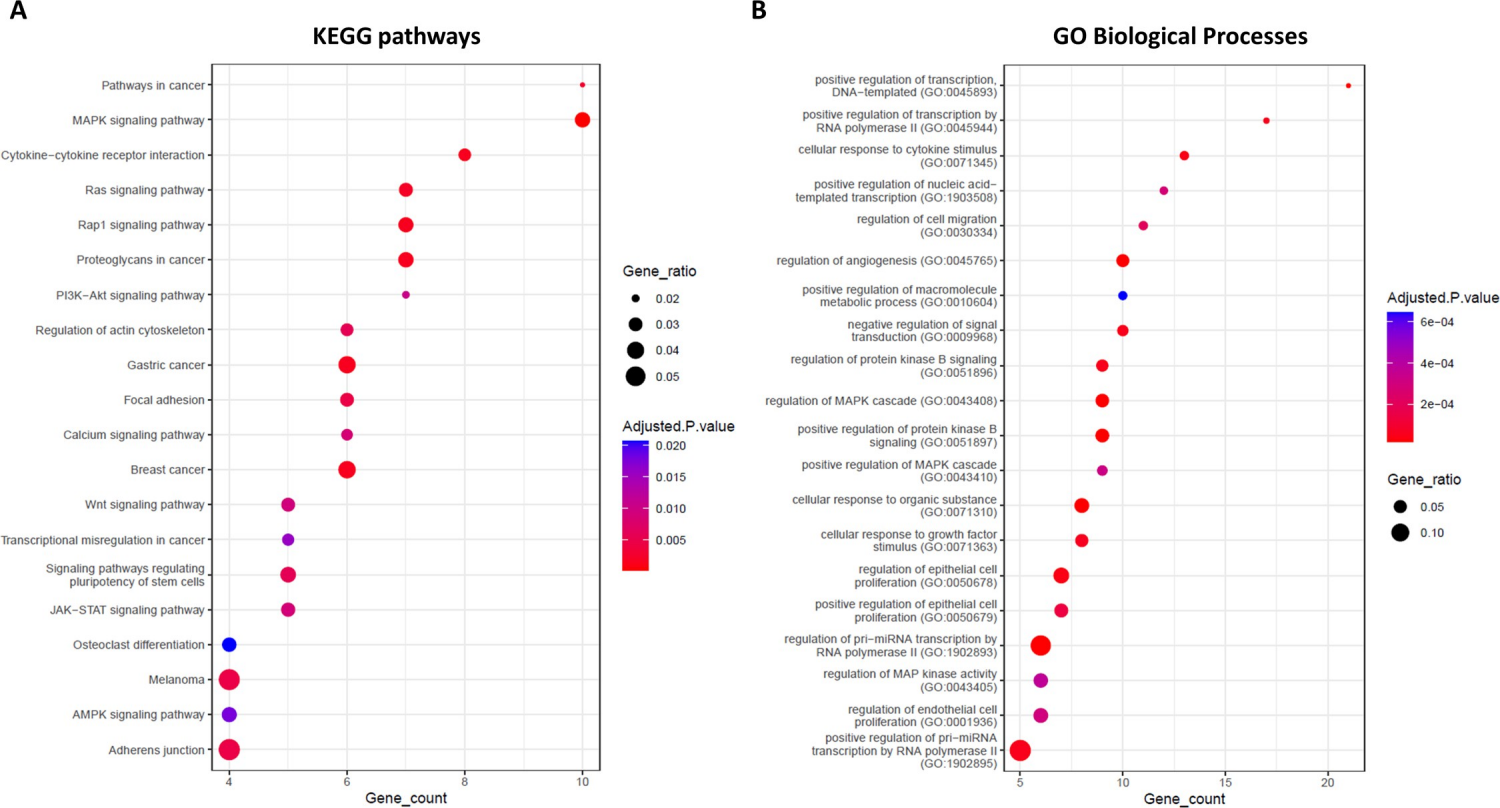
Enrichment analysis for first connected component of CERNOMA. Dot plot showing the top 20 KEGG pathways (A) and GO Biological Processes (B) (y axis) in which the mRNAs of the first connected component were enriched (adjusted p-value < 0.05) as function of the number of mRNAs found in each category (x axis). Nodes scale with the gene ratio (i.e., number of mRNAs over the total number of genes in that functional category) and are colored according to the adjusted p-value.

**Fig 6:**
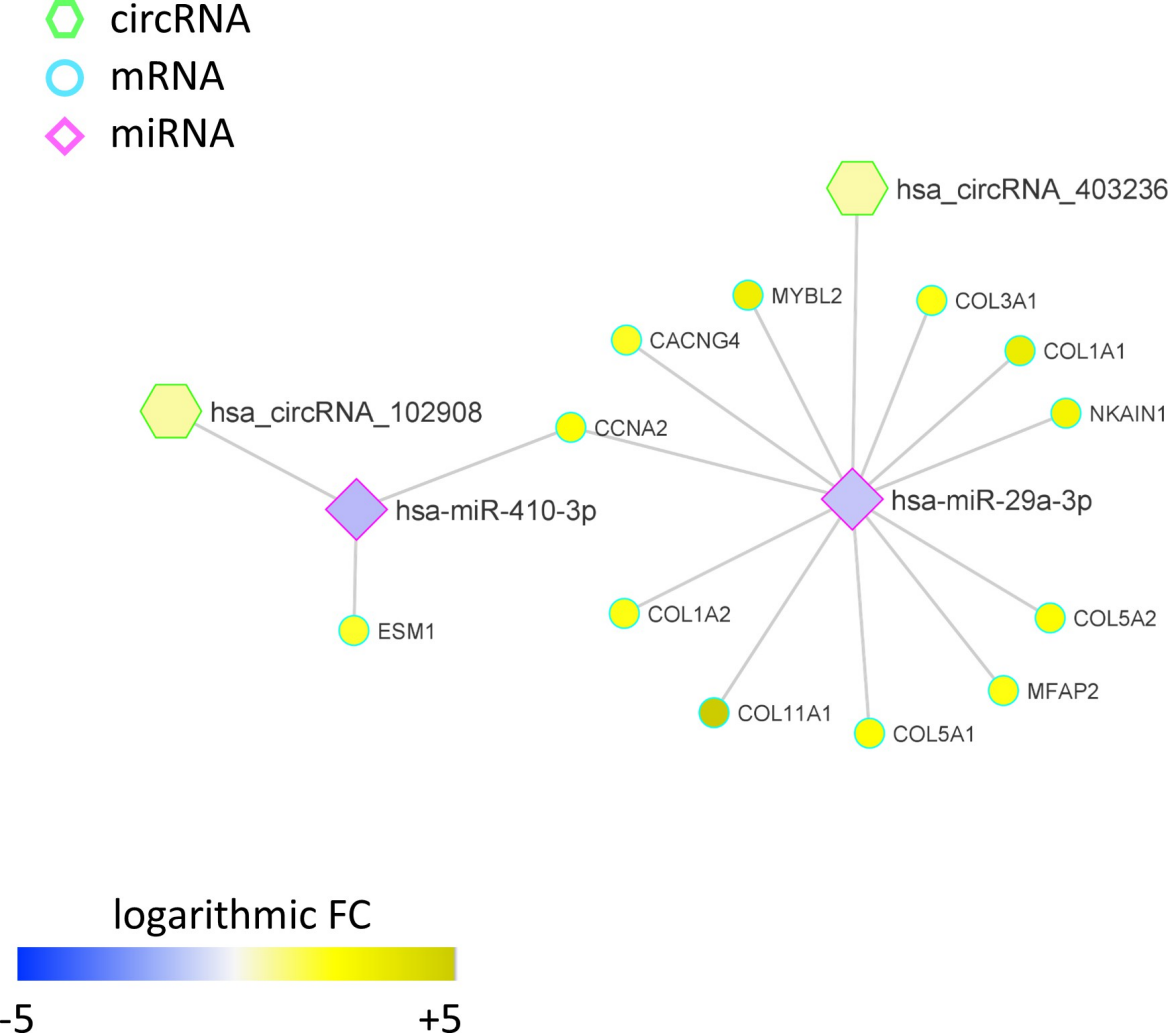
Second connected component of CERNOMA for breast cancer dataset. Network showing the circRNA-miRNA-mRNA interactions. Diamonds represents miRNAs, octagons represent circRNAs, circles represent mRNAs. Gradual changes in node color represent differences in the expression levels of different genes (increasing from blue to yellow).

**Fig 7:**
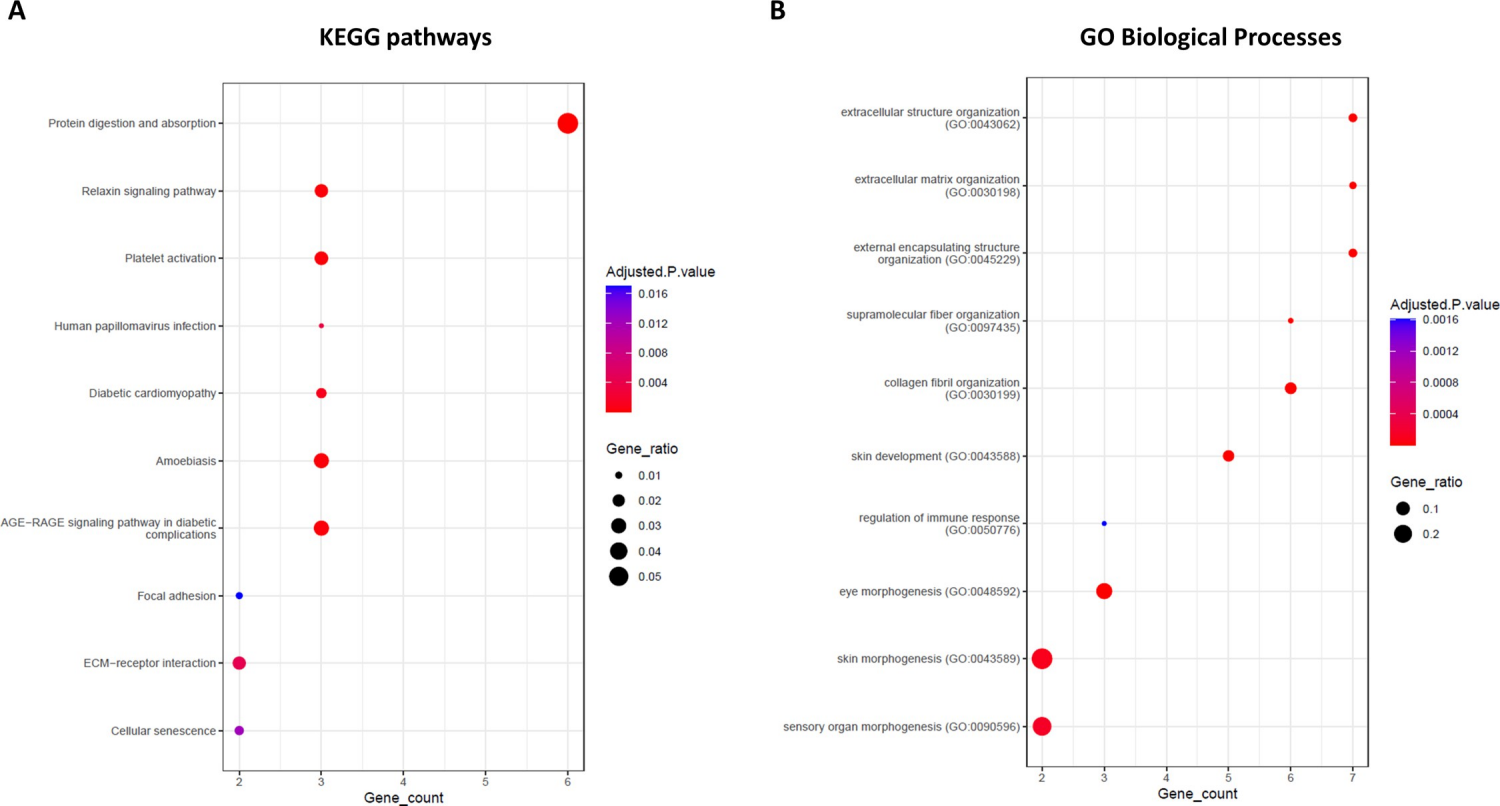
Enrichment analysis for second connected component of CERNOMA. Dot plot showing the top 10 KEGG pathways (A) and the GO Biological Processes (B) (y axis) in which the mRNAs of the second connected component were enriched (adjusted p-value < 0.05) as function of the number of mRNAs found in each category (x axis). Nodes scale with the gene ratio (i.e., number of mRNAs over the total number of genes in that functional category) and are colored according to the adjusted p-value.

Notably, the CERNOMA is marked by a clear segregation into two internally well-connected components (Figs 4 and 6), including genes involved in different pathways and biological processes (Figs 5 and 7). In particular, the first largest component (Fig 4) is composed of eight up-regulated miRNAs (hsa-miR-128-3p, hsa-miR-15a-5p, hsa-miR-15b-5p, hsa-miR-29a-3p, hsa-miR-200b-3p, hsa-miR-200c-3p, hsa-miR-301a-3p, hsa-miR-425-5p, hsa-miR-7-5p), two down-regulated circRNAs (hsa-circRNA-407041 and hsa-circRNA-104342), and 91 down-regulated mRNAs, mainly enriched in MAPK, PI3K, RAS, WTN, RAP1 signaling pathways, breast cancer pathway, cytokine-cytokine receptor interaction, and cytokine-related biological processes (S3 Table and Fig 5).

The smaller connected component (Fig 6) encompasses instead two down-regulated miRNAs (hsa-miR-410-3p and hsa-miR-29a-3p), two up-regulated circRNAs (hsa-circRNA-102908 and hsa-circRNA-403236), and involves 12 up-regulated mRNAs, mainly enriched in pathways and biological processes related to cell-cell communication, such as focal adhesion and extracellular matrix interaction (S3 Table and Fig 7).

## Discussion

CircRNAs are discovered as a special type of non-coding RNAs [3] likely plays a pivotal role in regulatory pathways controlling lineage determination, cell differentiation, and function of various cell types [20]. Due to their circular shape, circRNAs are resistant to degradation by exonuclease activity, making them more stable than linear RNAs and reliable biomarker. CircRNAs have been also revealed key players in diverse human cancers, functioning as regulator of the expression of their parental genes and exhibiting a ceRNA activity that may even affect disease [7, 8, 10, 12, 15, 22, 24]. Yet, the impact of circRNA-mediated regulation on various transcriptomes in cancer scenario still remains controversial and open-challenging field to explore [18].

In this study, we investigated the deregulation of circRNAs and their potential regulatory activity in human breast cancer via the development of a new computational pipeline, which first constructs a miRNA-target regulatory network composed of significantly deregulated circRNAs and mRNAs predicted to be target of significantly deregulated miRNAs; and then maps it onto a ceRNA space, ending up with the so-called CERNOMA, composed of circRNA-miRNA–mRNA triplets that could putatively exhibit RNA-RNA cross-talk activity. Within the released CERNOMA network, we can distinguish two connected components: the larger one including eight up-regulated miRNAs, two down-regulated circRNAs, and 91 down-regulated mRNAs (Fig 4); the smaller one including two down-regulated miRNAs, two up-regulated circRNAs, and 12 up-regulated mRNAs (Fig 6).

In the first connected component, we observe the down-regulated circular RNA hsa_circRNA_407041 showing a predicted binding site for: (i) two members of miR-200 family (hsa-miR-200b-3p and has-miR-200c-3p), whose deregulation have been already associated to human breast cancer development and progression [50–54]; (ii) hsa-miR-425-5p, whose overexpression has been recently observed to significantly promote breast cancer cell growth and predicted a poor prognosis for breast cancer patients [55]; (iii) hsa-miR-301a-3p, an oncogenic miRNA whose expression is associated with tumor development, metastases, and overall poor prognosis in breast cancer [56]; and (iv) hsa-miR-7-5p, which was already known to be inhibited by ciRS-7 [22] and whose over expression was found to be associated to poor prognosis in other cancers such as lung carcinomas [57]. We identified also the down-regulated hsa_circRNA_104342 showing a predicted binding for two members of miR-15 family (hsa-miR-15a-5p and hsa-miR-15b-5p), recently associated to breast cancer metastasis [58], and for hsa-miR-128-3p that has been shown to function as oncomiR in breast cancer tissues and cell lines, by increasing cell invasion, proliferation, and reducing apoptosis [59]. Downregulation of circRNAs is frequent in cancer cells, as observed in hepatocellular carcinoma, colorectal adenocarcinoma, prostate and ovarian cancer, lung adenocarcinoma [12]. In an attempt to better understand the potential role of molecular players in the disease development and progression, we studied the pathways and functional gene ontology (GO) processes in which they were involved. The KEGG pathway analysis indicated that the down-regulated DEGs of the first connected component were mainly associated with MAPK, PI3K, RAP1, WNT signaling pathways, breast cancer, cytokine-cytokine receptor interaction pathway (Fig 5A); and the GO analysis revealed that they were involved in cellular response to cytokine stimulus, positive regulation of transcription, regulation of Mitogen-Activated Protein Kinase (MAPK) cascades (Fig 5B). MAPK pathway is evolutionarily conserved kinase module, which participates in several intracellular signaling pathways and plays an important role in controlling a wide spectrum of cellular processes, including proliferation, growth, migration, differentiation, and apoptosis. Abnormal functioning of MAPK signaling pathways can play a crucial role in cancer development and progression [60, 61].

In the second connected component, we can observe the up-regulated circular RNA hsa_circRNA_102908 showing a predicted binding for hsa-miR-410-3p, which has been predicted to bind also the up-regulated ESM1 gene. Increased expression level of ESM1 has been shown to exhibited significantly enhanced proliferation, migration, and invasion in breast cancer cells [10], as well as an aberrant expression of hsa-miR-410-3p is common in a variety of cancers including breast cancer, suggesting that miR-410-3p may play an important role in cancer development and progression [21]. The circular RNA hsa_circRNA_102908 is originated from the BRCA1 associated RING domain 1 and has been already found significantly up-regulated in human radioresistant esophageal cancer cell line KYSE-150R when compared with the parental cell line KYSE-150, suggesting its possible involvement in the development of radiation resistance and treatment failure [62]. We also found the up-regulated circular RNA hsa_circRNA_403236 predicted to bind hsa-miR-29a-3p, which in turn could target several genes encoding for the collagens family of proteins that strengthen and support many tissues. Cell-cell adhesion is well-known to be a fundamental process for tissue architecture and morphogenesis, and its alteration can disrupt important cellular processes and lead to a variety of diseases, including cancer [63]. Both KEGG and GO functional analyses confirmed that the up-regulated DEGs of the second connected component were mainly enriched in cell communication processes, such as extracellular matrix interaction and focal adhesion pathways (Fig 7A), as well as extracellular matrix organization and structure biological processes (Fig 7B).

The analysis conducted in this study can be generalized to investigate other pathologies and could offer potential insights for the disease understanding that are worthy of further investigation.

## Supporting information

S1 Table Differentially expressed genes. This table lists, in three separated sheets, the differentially expressed RNAs (DEGs), miRNAs (DEMs), and circRNAs (DECs), respectively.
(XLSX)

S2 Table ceRNA network. This table includes the miRNA-target regulatory network (MRN), the CERNOMA, and the list of all the circRNA-miRNA-mRNA triplets forming the CERNOMA together with their statistics obtained for breast cancer dataset.
(XLSX)

S3 Table Functional enrichment analysis. This table includes the results of functional enrichment analysis for the DEGs included in the first and second component of the CERNOMA.
(XLSX)
